# Multi-Omics Integration Reveals PBDE-47 as an Environmental Risk Factor for Intracranial Aneurysm via F2R-Mediated Metabolic and Epigenetic Pathways

**DOI:** 10.3390/brainsci15101091

**Published:** 2025-10-09

**Authors:** Hongjun Liu, Jinliang You, Junsheng Bai, Dilaware Khan, Sajjad Muhammad

**Affiliations:** 1 Department of Neurosurgery, Medical Faculty, University Hospital Düsseldorf, Heinrich-Heine-Universität, 40225 Düsseldorf, Germany; liuhongjun321@nsmc.edu.cn (H.L.); you.jinlian@med.uni-duesseldorf.de (J.Y.); junshengbai.med@gmail.com (J.B.); dilaware00@yahoo.com (D.K.); 2 Department of Neurosurgery, The Affiliated Hospital of North Sichuan Medical College, Nanchong 637000, China

**Keywords:** PBDE-47, environmental pollutant mechanisms, intracranial aneurysm, F2R, metabolic-epigenetic regulation

## Abstract

**Background:** Intracranial aneurysm (IA) rupture is a life-threatening cerebrovascular event with a mortality rate of up to 40%, affecting approximately 500,000 people globally each year. Although environmental pollutants such as 2,2′,4,4′-tetrabromodiphenyl ether (PBDE-47) have been implicated in the pathogenesis of IA, the causal relationship and underlying mechanisms remain unclear. This study aims to systematically explore the potential causal role of PBDE-47 in the development of IA by integrating multi-omics approaches. **Methods:** We utilized the UK Biobank Drug Proteomics Project (UKB-PPP) genome-wide association study (GWAS) data, including 2940 plasma proteins and 1400 metabolites, along with IA genetic data from 456,348 individuals, to perform a two-sample Mendelian randomization (MR) analysis. Instrumental variables were selected based on genome-wide significance (*p* < 5 × 10^−8^) or suggestive thresholds (*p* < 5 × 10^−5^). Analytical methods included inverse variance weighting (IVW), MR-Egger, weighted median, MR-PRESSO, and Steiger filtering for sensitivity analysis. Molecular docking and 100-nanosecond molecular dynamics simulations were used to evaluate interactions between PBDE-47 and proteins. Mediation analysis assessed the roles of plasma metabolites and miRNAs, and SMR-HEIDI tests were used to verify causal relationships. **Results:** MR analysis identified 93 plasma proteins potentially causally associated with IA, including 53 protective factors and 40 risk factors. By integrating PBDE-47 targets, IA-related genes, and metabolite-related genes, we identified 15 hub genes. Molecular docking revealed potential binding between PBDE-47 and F2R (binding energy: −5.516 kcal/mol), and SMR-HEIDI testing supported F2R as a potential causal risk factor for IA. Molecular dynamics simulations indicated the stability of the complex structure. Mediation analysis suggested that F2R may influence IA risk through eight plasma metabolites, and miR-130b-3p may indirectly promote IA development by upregulating F2R. **Conclusions:** Our findings suggest that exposure to PBDE-47 may have a potential causal relationship with IA risk, potentially mediated through the “PBDE–47–F2R–metabolite–miRNA” regulatory axis. These results provide preliminary evidence for early diagnostic biomarkers and targeted interventions for IA. The multi-omics analytical framework established in this study offers new insights into environmental determinants of neurovascular diseases, although further validation is needed to address potential limitations.

## 1. Introduction

Intracranial Aneurysms are a serious cerebrovascular disorder characterized by structural defects in the tunica media of arteries, leading to vessel wall weakening and aneurysm formation. The global prevalence of IA is estimated at 1–5% among adults aged 35–75 [1], with aneurysm rupture causing nearly 500,000 cases of subarachnoid hemorrhage (SAH) annually. This results in a 30-day mortality rate of approximately 40%, with many survivors suffering from permanent neurological deficits [2]. Despite advances in diagnostic techniques, such as CT, CTA, MRI, and DSA, there are currently no reliable early biomarkers or preventive treatments for IA, highlighting the need for improved molecular insights into its development [3,4].

The pathogenesis of IA remains incompletely understood, particularly regarding the role of environmental factors. Recently, growing attention has been directed towards environmental pollutants as potential contributors to cerebrovascular diseases [5]. One such pollutant is the flame retardant PBDE-47, which is known to have neurotoxic effects and disrupt vascular homeostasis [6,7]. However, its specific link to IA remains underexplored. Studies have shown that PBDE-47 induces vascular toxicity, alters metabolomic and lipidomic profiles [8,9], and influences key cellular pathways, suggesting its potential involvement in the pathogenesis of IA. We hypothesize that PBDE-47 exposure could disrupt the extracellular matrix (ECM) in brain-specific arteries, a critical component of vascular architecture that plays a vital role in maintaining arterial wall integrity.

The ECM is crucial in maintaining the structural integrity of blood vessels, especially in the cerebral vasculature. Disruption of the ECM leads to vascular remodeling, loss of vascular smooth muscle cells, and increased inflammation, which are critical factors in aneurysm formation and rupture [10]. Given PBDE-47’s known effects on vascular toxicity, we propose that its interaction with the ECM could be central to the development of IA by promoting endothelial dysfunction and vascular instability, key contributors to aneurysm formation.

Despite these promising implications, the evidence supporting the role of PBDE-47 in IA remains sparse. Current research faces challenges, such as the difficulty of eliminating confounding variables in traditional observational studies, and the inability of isolated cell culture models to fully replicate the complexities of human biology. Moreover, environmental pollutants like PBDE-47 often target multiple biological pathways, complicating efforts to decipher their precise molecular mechanisms in diseases like IA.

To address these gaps, our study employs a multi-omics integrative analysis, combining Mendelian randomization, molecular docking, and molecular dynamics simulations. This approach provides several advantages: (1) Mendelian randomization allows us to control for confounding biases and establish causal relationships between PBDE-47 exposure and IA; (2) Molecular simulations offer detailed atomic-level insights into the interactions between PBDE-47 and vascular proteins involved in ECM remodeling; and (3) Multi-omics integration reveals the entire sequence from exposure to regulation, reprogramming, and pathology. These tools will help us understand how PBDE-47 contributes to IA through its effects on the ECM and other vascular processes.

Ultimately, this study aims to determine whether PBDE-47 exposure disrupts the ECM in brain-specific arteries, contributing to IA development. By examining how environmental factors like PBDE-47 influence IA, we hope to provide new insights into the molecular mechanisms of IA and identify potential targets for early detection and prevention.

## 2. Methods

### 2.1. Study Design

**Figure 1:**
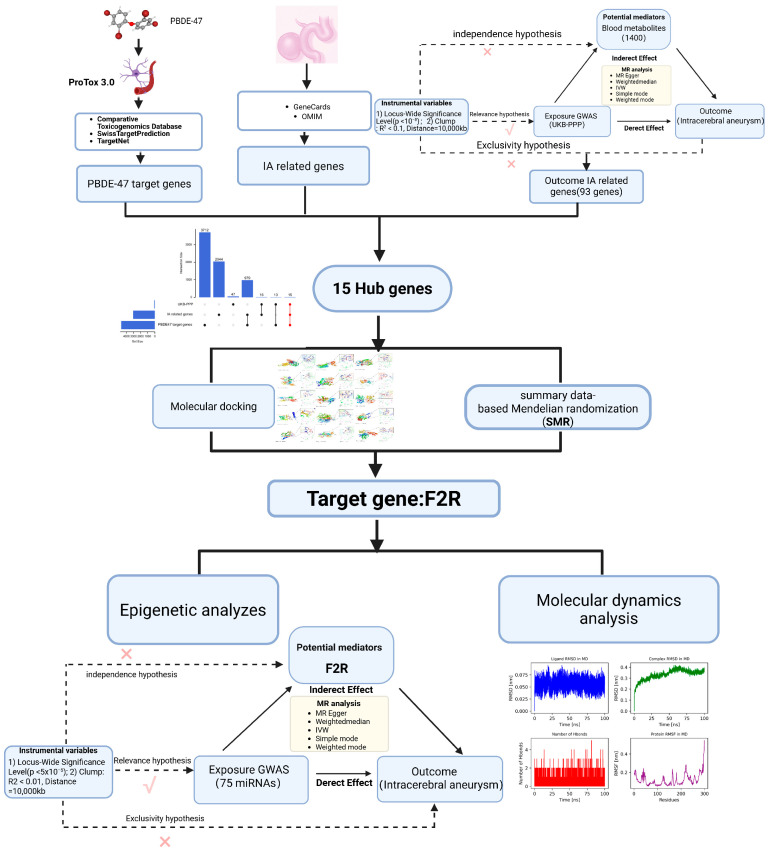
The workflow of research analysis.

Figure 1 presents the analytical workflow of this study, employing a multi-omics integration strategy to systematically investigate how PBDE-47 exposure leads to IA. First, we constructed a two-stage Mendelian randomization model based on the mQTL data of 1400 plasma metabolites from the UKB-PPP project. In the first stage, we established the association between UKB-PPP genes and metabolites. we evaluated the causal influences of metabolites on IA through the application of inverse variance weighting (IVW) and Mendelian Randomization Pleiotropy RESidual Sum and Outlier (MR-PRESSO). To ensure the reliability of the mediation effects observed, we employed Steiger directional tests, combined likelihood ratio tests (CLR), and Bootstrap techniques with 1000 iterations. Next, we predicted the ADME properties and toxicity of PBDE-47 using ProTox3.0 (https://tox.charite.de/protox3/index.php?site=home), accessed on 7 June 2025. Then, we identified candidate genes for IA using the GenCards and OMIM databases. Furthermore, by intersecting target genes of PBDE-47, IA risk genes, and genes related to mediating metabolites, we identified 15 hub genes. Through molecular docking (binding energy ≤ −5 kcal/mol) and applying SMR-HEIDI tests, we ultimately identified the core target F2R. Finally, we performed 100 ns molecular dynamics simulations (RMSD < 0.2 nm) to reveal the interaction pattern between PBDE-47 and F2R. We also constructed a regulatory network that includes miRNA, F2R, plasma metabolites, and IA. Using FDR multiple testing correction (*p* < 0.05) and leave-one-out sensitivity analysis, we ensured the reliability of the results, comprehensively demonstrating the multi-dimensional mechanism pathway of “chemical exposure-metabolic reprogramming-epigenetic regulation-disease occurrence”. This research was carried out in compliance with the Declaration of Helsinki, as amended in 2013.

### 2.2. Data Sources

Plasma protein data were sourced from the UK Biobank-Proteomics Project (UKB-PPP) [10,11,12], which contains Genome-Wide Association Studies (GWAS) data for 2940 plasma proteins. In 2023, GWAS data for 1400 plasma metabolites were acquired from a study in the GWAS database (identifier range: GCST90199621 to GCST90201020) (Supplementary Table S2), focusing on European populations [13]. Genetic information related to IA was sourced from the GWAS database, using dataset identifier GCST90044003, which includes 456,348 samples and 11,842,647 SNPs from European populations [14]. miRNA eQTL data were collected from a whole-blood study of 5239 individuals, which identified 5269 cis miR-eQTLs associated with 76 mature miRNAs [15]. All data sources are listed in Supplementary Table S1.

### 2.3. Instrumental Variable Selection

In order to assess causal effects arising from genetic variations, it is essential that three primary assumptions of instrumental variables (IV) analysis are satisfied: (1) The IV must relate to the exposure factor; (2) The IV must be free from confounding factors; (3) The IV must affect the outcome solely through the exposure factor [16,17,18]. The IVs chosen for this study followed these criteria: (1) If the number of genome-wide significant loci was limited to 35 or fewer, we selected SNPs that met either a locus-specific threshold (*p* < 5 × 10^−8^) or genome-wide significance (*p* < 5 × 10^−5^) as potential instruments. (2) We excluded SNPs showing association with the outcome (*p* < 0.05). (3) We conducted clumping to reduce linkage disequilibrium effects, setting parameters of r^2^ < 0.1 with a window size of 500 kb, or r^2^ < 0.01 with a window size of 10,000 kb. (4) MR-PRESSO detected and removed outliers with horizontal pleiotropy, sequentially eliminating SNPs until the global test *p* > 0.05 [19,20]. (5) Steiger filtering removed IVs showing stronger outcome than exposure associations.

In this study, to assess the causal effects of genetic variations on exposure factors, we strictly adhered to the three core assumptions of Mendelian Randomization (MR) analysis: (1) the instrumental variable (IV) must be related to the exposure, (2) the IV must be free from confounding, and (3) the IV must affect the outcome solely through the exposure. SNPs were selected as instrumental variables based on the following criteria: for genome-wide significant SNPs, the traditional significance threshold (*p* < 5 × 10^−8^) was applied; for exposures with a limited number of significant SNPs, a more relaxed threshold (*p* < 5 × 10^−5^) was used. This approach of appropriately relaxing the IV selection threshold (e.g., *p* < 5 × 10^−5^) has been widely adopted in previous studies [21,22]. To minimize potential bias and pleiotropy, we employed a multi-step process: First, SNPs associated with the outcome (*p* < 0.05) were excluded; second, clumping was performed to remove linkage disequilibrium (LD), selecting independent SNPs based on r^2^ values (<0.1 within a 500 kb window or <0.01 within a 10,000 kb window); third, MR-PRESSO was applied to detect and remove SNPs exhibiting horizontal pleiotropy, iteratively eliminating outliers until the global test *p*-value was >0.05; finally, Steiger filtering was used to exclude SNPs with stronger associations with the outcome than with the exposure, ensuring correct causal direction. Through these rigorous selection steps, we ensured that the chosen IVs satisfied the key assumptions of MR analysis, effectively mitigating pleiotropy and confounding bias.

### 2.4. Reverse Causal Analysis

We performed a reverse Mendelian randomization analysis where metabolites served as the exposure variables and significant proteins were designated as the outcome measures. Steiger filtering served as an alternative directional test, excluding SNPs where outcome associations surpassed exposure associations, indicating probable non-causal relationships.

### 2.5. Collection of PBDE-47 Targets

The chemical composition and SMILES representation of PBDE-47 were sourced from PubChem (http://www.ebi.ac.uk/chembl/). To assess the toxicity profile of PBDE-47, we employed Protox-3.0. Subsequently, we identified possible targets for PBDE-47 through the Comparative Toxicogenomics Database (CTD) (https://ctdbase.org/), SwissTargetPrediction (http://www.swisstargetprediction.ch/), and TargetNet (http://targetnet.scbdd.com/). Following this, we utilized STRING (https://string-db.org/) and the Universal Protein Resource (UniProt) (https://www.uniprot.org/) to ensure the standardization of the target nomenclature. Ultimately, we established a database of PBDE-47 targets by integrating and refining the identified targets.

### 2.6. Retrieval of IA Targets

Utilizing “Intracranial aneurysm” as the keyword for our search, pertinent targets were obtained from both the GeneCards (https://www.genecards.org/) and OMIM (https://www.omim.org/) databases. Subsequently, these targets were consolidated into a comprehensive dataset pertaining to IA targets. The criteria for screening included a relevance score from GeneCards that was greater than or equal to 1. (All dates are accessed on 7 June 2025).

### 2.7. Molecular Docking

Molecular docking methods were employed to investigate the interaction between PBDE-47 and the primary target. The process involved several steps: First, the SMILES structure of PBDE-47 was obtained from the PubChem database. Next, the crystal structure of the target protein was downloaded from the PDB database using the UniProtID. The protein receptor was preprocessed with PyMol 2.5 by removing water molecules, salt ions, and non-essential ligands [23]. Subsequently, D3pocket was used to predict the active pocket, and a docking box of 30 Å was set with the pocket center as the reference [24]. Semi-flexible docking was performed using AutoDockVina1.2.0 [25], and the molecules and receptors were converted to PDBQT format using ADFRsuite. In order to assess the reliability of the method, a re-docking experiment was performed utilizing the original crystal ligand, achieving a root-mean-square deviation (RMSD) of under 2 Å. This outcome serves to validate the effectiveness of the approach [26]. Ultimately, the conformation exhibiting the minimal Vina binding energy was chosen, and critical interactions—including hydrogen bonds, hydrophobic interactions, and van der Waals forces—were examined utilizing the visualization capabilities of Discovery Studio (Version: Discovery Studio 2025 Client). 2D binding mode diagram was generated to elucidate the molecular recognition mechanism.

### 2.8. Molecular Dynamics (MD) Simulation

We performed MD simulations utilizing the AMBER22 software package (version 22.0) [27], starting from a structure formed by the docked complex of the small molecule and protein. Before the simulation, we calculated the small molecule’s partial charges using the antechamber module and Gaussian09 software [28,29]. We employed the Hartree–Fock (HF) SCF/6-31G theory for this calculation. We parameterized the small molecule with the GAFF2 force field and the protein with the ff14SB force field. Using the LEaP module, we added hydrogen atoms to each system and then solvated them in a truncated octahedral TIP3P water box that extended 10 Å beyond the system. Na^+^/Cl^−^ ions were added to neutralize the system charge, and the final topology and parameter files were generated for simulation.

The molecular dynamics simulations were conducted in AMBER22. The energy minimization of the system was executed through a sequence of 2500 steps utilizing the steepest descent method, which was then succeeded by an additional 2500 steps applying the conjugate gradient technique. Following this, the system was subjected to a heating phase lasting 200 picoseconds under NVT (canonical ensemble) conditions, during which the temperature was incrementally raised from absolute zero (0 K) to 298.15 K. To achieve a consistent distribution of the solvent, a subsequent equilibration phase of 500 picoseconds under NVT conditions was conducted. Next, we performed a 500 ps equilibration under NPT (isothermal-isobaric ensemble) conditions. Finally, a 100 ns production simulation was carried out under NPT conditions with periodic boundary constraints. A cutoff distance of 10 Å was established for non-bonded interactions, while long-range electrostatic interactions were computed employing the Particle Mesh Ewald (PME) technique [30]. Hydrogen bond lengths were constrained using the SHAKE algorithm [31], while temperature was regulated via the Langevin thermostat with a collision frequency (γ) of 2 ps^−1^ [32]. The system pressure was maintained at 1 atm, with a 2 fs integration time step and trajectory frames saved every 10 ps for analysis.

### 2.9. MM/GBSA Binding Free Energy Calculation

The binding free energy between the protein and ligand in all systems was calculated using the MM/GBSA method [33,34,35]. The MD trajectories from 90 to 100 ns were used for the calculations, the specific equation is expressed as follows:

ΔG_bind = ΔG_complex − (ΔG_receptor + ΔG_ligand)  = ΔE_internal + ΔE_VDW + ΔE_elec + ΔG_GB + ΔG_SA(1)

In Equation (1), ΔE_internal denotes the internal energy, ΔE_VDW signifies the van der Waals interactions, and ΔE_elec indicates the electrostatic interactions. The internal energy encompasses bond energy (E_bond), angular energy (E_angle), and torsional energy (E_torsion). The terms ΔG_GB and ΔG_SA are collectively referred to as solvation free energy, where ΔG_GB corresponds to the polar solvation free energy, while ΔG_SA pertains to the nonpolar solvation free energy. The GB model utilized for ΔG_GB was developed by Nguyen et al. [36]. For the calculations, an igb value of 2 was employed. The nonpolar solvation free energy (ΔG_SA) was determined using the equation ΔG_SA = 0.0072 × ΔSASA, which incorporates the product of surface tension (γ) and the solvent-accessible surface area (SA) [37]. Entropy changes were ignored in this study due to their high computational cost and low accuracy.

### 2.10. Statistical Analysis

We used a MR framework to systematically assess how plasma proteins and microRNAs (miRNAs) causally influence IA and its underlying mechanisms. The IVW method served as the primary analytical approach, supplemented by MR-Egger regression and weighted median methods for causal inference. Sensitivity analyses, including Cochran’s Q test, MR-PRESSO, and leave-one-out analysis, confirmed the validity of instrumental variable assumptions (no heterogeneity or horizontal pleiotropy, *p* > 0.05). We assessed mediation effects using the product-of-coefficients method, where β_1_ represents the effect of the exposure on the mediator, β_2_ represents the effect of the mediator on IA, and the indirect effect is calculated as β_1_ × β_2_. SMR [38]-HEIDI [39] validation further supported the causal relationships between gene expression and IA, with SMR showing significance (*p* < 0.05) and HEIDI indicating no significant heterogeneity (*p* > 0.05). All analyses were conducted using the R package TwoSampleMR (v0.6.0) and SMR software (v1.3.1), with statistical significance defined as IVW *p* < 0.05.

## 3. Results

### 3.1. Genetic Causal Relationship Between UKB-PPP and IA: MR Analysis

This study used a two-step MR framework to systematically assess how plasma proteins from the UKB-PPP affect IA. We incorporated five distinct causal inference methodologies—namely, IVW, Simple Median, Weighted Median, Weighted Mode, and MR-Egger regression—into a unified validation framework [40,41,42]. Using a strict multiple testing correction with a significance level of *p* < 0.05, the IVW analysis identified 93 plasma proteins that have significant causal associations with IA (Supplementary Table S3). Of these proteins, 53 demonstrated significant negative causal associations, indicating protective effects—examples include ACAA1 (OR = 0.2190, 95% CI: 0.0743–0.6455) and ACP5 (OR = 1.4267, 95% CI: 1.0414–1.9546). Conversely, 40 proteins, such as FGFR2 (OR = 3.0938, 95% CI: 1.3835–6.9188) and FGL1 (OR = 1.2216, 95% CI: 1.0075–1.4813), exhibited positive causal associations, suggesting risk effects (Supplementary Figure S1A). The bidirectional MR analysis showed significant reverse causal relationships for ACP5 (OR = 1.0043, 95% CI: 1.0006–1.0081) and CCS (OR = 0.9957, 95% CI: 0.9917–0.9997), indicating that these proteins may be regulated by the status of IA disease. Consequently, they were excluded from further mechanism analyses (Supplementary Figure S1B, Supplementary Table S4).

### 3.2. PBDE-47 Toxicity and Target Prediction

**Figure 2:**
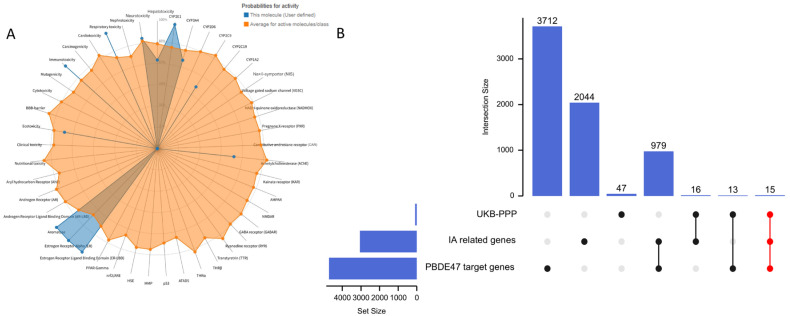
(**A**) Predicted toxicological effects and metabolic activation of PBDE-47; (**B**) Venn diagram illustrating the intersection of molecular targets associated with PBDE-47 exposure and IA pathogenesis.

We first utilized the ProTox-3.0 toxicity prediction platform to conduct a comprehensive assessment of PBDE-47’s toxicological profile. The results revealed that this compound exhibits significant immunotoxicity (prediction probability: 0.96), estrogen receptor α (ERα) activation (prediction probability: 0.99), and respiratory toxicity (prediction probability: 0.98). These toxicological characteristics suggest that PBDE-47 may contribute to vascular pathology through multiple mechanisms: (1) promoting inflammatory factor release via immune system activation; (2) disrupting vascular homeostasis through the ERα signaling pathway; (3) causing vascular endothelial damage through oxidative stress induction. PBDE-47’s metabolic activation mainly relies on the CYP3A4 (probability: 0.71) and CYP2C9 (probability: 0.56) enzymes. This process may produce harmful intermediates that affect blood vessels (Figure 2A).

For molecular target identification, we established a systematic screening protocol: (1) retrieving 4655 PBDE-47 targets from the Comparative Toxicogenomics Database (CTD); (2) supplementing with 100 high-confidence targets from SwissTargetPrediction; (3) collecting 18 experimentally validated interaction targets from ChEMBL. After thorough data cleaning and removal of duplicates, we identified 4719 molecular targets related to PBDE-47. Concurrently, by integrating GeneCards (2931 IA-related targets) and OMIM (200IA-related targets) databases and removing duplicates, we established a set of 3110 IA-associated targets. Additionally, we included 91 genes showing significant causal associations with IA. By conducting a systems biology analysis that integrated these three target datasets, we identified 15 common targets (CTSS, CTSC, CRH, ADIPOQ, ANGPTL4, CAT, F2R, CCDC80, CDH17, COL9A1, CSF3, CXCL11, F7, FGF5, FGFR2) that connect PBDE-47 exposure to the pathogenesis of IA (Figure 2B).

### 3.3. Molecular Docking Analysis

**Figure 3:**
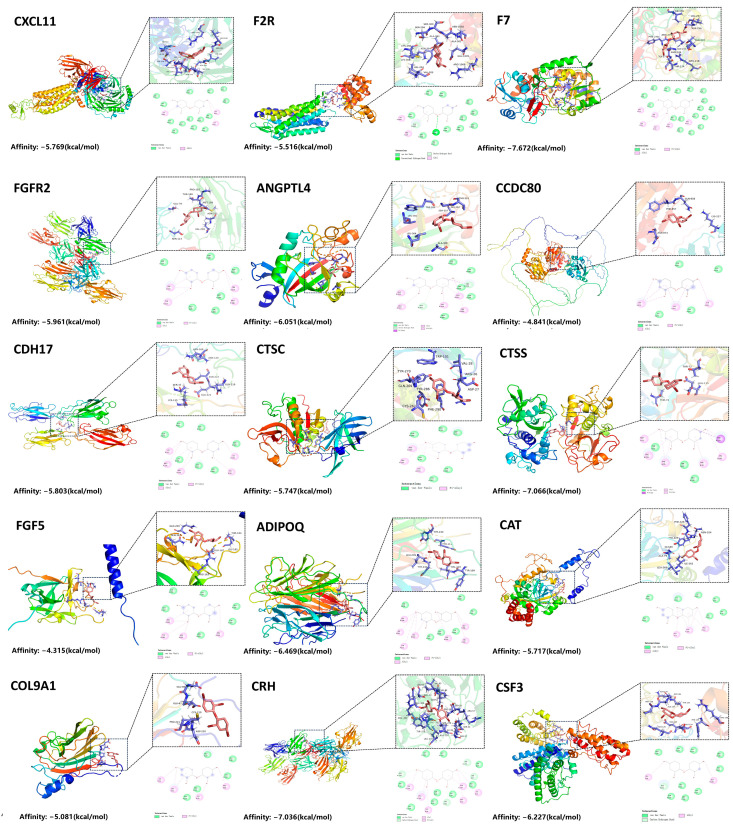
Molecular Docking of PBDE-47 with Target Proteins.

This research employed molecular docking methodologies to evaluate the binding affinity of PBDE-47 with 15 disease-associated targets, thereby confirming its potential toxicological effects (Figure 3). We retrieved the crystal structures of these targets from the Protein Data Bank (PDB). The strength of the interactions was quantified using binding free energy (ΔG) calculations, where more negative ΔG values indicate a stronger binding affinity. The results indicated that PBDE-47 formed stable complexes with all targets (Table 1). The lowest binding energy was observed for the interaction with coagulation factor F7 (−7.872 kcal/mol), suggesting the strongest affinity. These findings suggest that PBDE-47may interfere with biological functions by specifically targeting and binding critical proteins such as F7, CRH, and CTSS, thereby causing toxic effects. In light of the findings presented, we propose that the proteins identified as targets are instrumental in the onset and advancement of IA induced by PBDE-47.

### 3.4. The F2R cis-pQTL Demonstrated Its Reliability by Passing Both the SMR and HEIDI Tests

In this study, we selected 13 proteins with binding energies < −5 kcal/mol for summary-data-based Mendelian randomization (SMR) analysis to investigate their potential causal relationships with IA. To ensure accuracy and reliability, we integrated various genetic data sources, Including GWAS, expression quantitative trait loci (eQTL), and LD reference datasets. Subsequently, we performed an extensive evaluation and examination of cis-eQTLs.

The analysis included 11,831,932 single nucleotide polymorphisms (SNPs), ensuring clarity in our methodology. Following checks for allele frequency consistency, we excluded 4627 SNPs that had a difference threshold greater than 0.20. Ultimately, six probes detected significant cis-eQTL signals (*p* < 5.00 × 10^−8^). To further refine the data, we performed LD filtering, removing redundant loci with r^2^ > 0.90 or <0.05. The cis-protein quantitative trait locus (cis-pQTL) of F2R successfully passed both SMR (*p* < 0.05) and HEIDI (*p* > 0.05) tests, indicating no heterogeneity in instrumental variables and robust causal associations. These results suggest F2R may be a potential risk factor for aneurysm formation (Table S5).

### 3.5. MD Simulation Analysis

**Figure 4:**
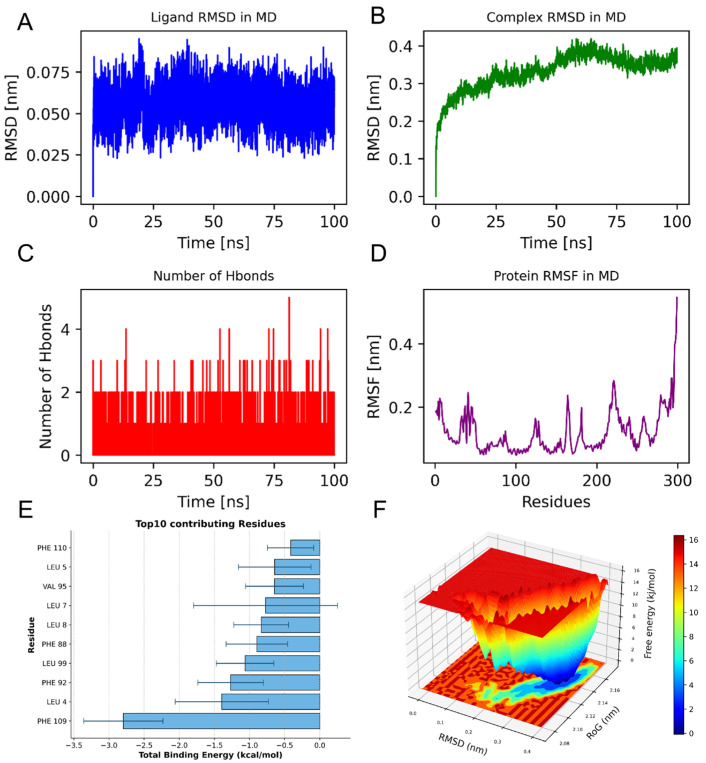
(**A**) PBDE-47_F2R complex stability analysis; (**B**) RMSD evolution of PBDE-47_F2R complex; (**C**) Hydrogen bond dynamics during simulation; (**D**) Residue-specific flexibility (RMSF) of F2R; (**E**) The ten amino acids that significantly influence the binding energy are as follows: (**F**) Free energy landscape (RMSD vs. Rg) showing stable conformation at 0.35 nm/2.14 nm.

We conducted MD simulations to validate the binding capability of small-molecule compounds with the key target protein. We identified F2R as a potential risk factor for aneurysm development based on its strong binding affinity in molecular docking, successful validation through SMR and HEIDI tests, and—crucially, despite F7 exhibiting lower binding energy—its superior supporting evidence from genetic causal inference and central position in a biologically coherent pathway that integrates miRNA regulation with metabolic mediation. Therefore, we selected the PBDE-47/F2R protein complex for MD simulations. The equilibrium condition of the simulated system was assessed through the application of root-mean-square deviation (RMSD) analysis. This involved measuring the RMSD for both the ligand and the ligand-protein complex throughout the simulation period, tracking the dynamics of hydrogen bonds formed between the ligand and the protein over time, as well as evaluating the RMSF profile of the protein. Analysis of the RMSD changes in the ligand in the F2R complex shows that the ligand’s RMSD fluctuates slightly and remains stable at approximately 0.05 nm throughout the simulation Figure 4A. Meanwhile, the overall RMSD of the complex increases from its initial value and fluctuates around 0.4 nm during the simulation, indicating the overall stability of the complex Figure 4B. This trend suggests that the binding of the ligand does not significantly disrupt the global conformation of F2R. The hydrogen bond analysis indicates that the number of hydrogen bonds between the ligand and the receptor remains stable throughout the simulation, averaging about one bond. This further supports the stability of the ligand-receptor interaction Figure 4C. These results indicate that the ligand can effectively bind and stably exist at the binding site of F2R. Combined with RMSF analysis, the overall RMSF values of the protein’s amino acids are low, below 0.5 nm, indicating that the rigidity of the binding site is stronger, which helps to maintain the stability of ligand binding Figure 4D.

The analysis of binding energy contributions identifies PHE 109 as the key residue contributing the most to ligand binding, with a value of approximately −2.8 kcal/mol. This residue likely forms hydrophobic interactions that stabilize the binding of the ligand. In addition, residues such as LEU-4, PHE-92, and LEU-99 also significantly contribute to the total binding energy, indicating that these residues are the main areas for forming stable binding Figure 4E.

### 3.6. Molecular Mechanics, Generalized Born, Surface Area MM-GBSA Results

Using molecular dynamics simulations, we applied the MM-GBSA method to calculate the binding energy, which more accurately reflects the interaction between the small molecule and the target protein. As shown in Table 2, The binding energy of F2R_PBDE-47 is −37.85 ± 3.12 kcal/mol. A negative value indicates that the molecules bind to the target protein, with lower values signifying stronger binding. Our calculations indicate that the binding affinity of F2R_PBDE-47 is strong. Energy decomposition shows that the primary contributions to the binding of F2R_PBDE-47 are from van der Waals energy, electrostatic energy, and nonpolar solvation free energy.

The free energy surface (FEL) results of the F2R and PBDE-47 complex are presented below. The figure uses color mapping to represent different free energy values, with the X-axis representing RMSD in nanometers (nm), and the Y-axis representing Rg (Radius of Gyration), also in nanometers. Red areas in the figure indicate higher free energy, while blue areas indicate lower free energy. Through this figure, it can be observed that the complex has a lower free energy at RMSD equal to 0.35 nm and Rg equal to 2.14 nm, indicating that the complex possesses a global minimum energy point and that the complex structure is stable Figure 4F.

### 3.7. F2R Mediates IA Risk Through Plasma Metabolites

**Figure 5:**
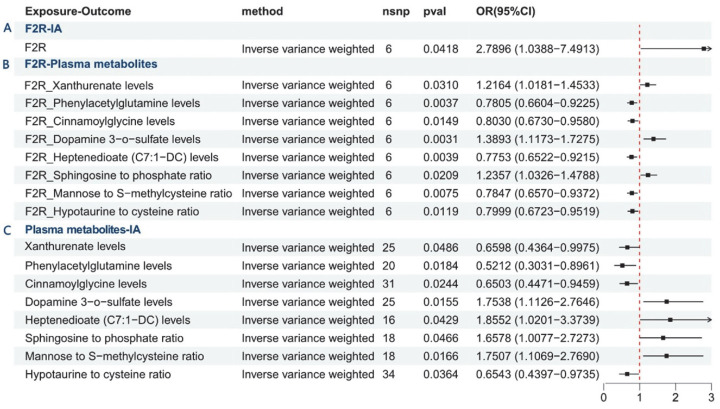
Mendelian randomization analysis of the causal relationship between F2R, plasma metabolites, and IA. (**A**) F2R shows a significant positive causal effect on IA risk; (**B**) Regulatory effects of F2R on plasma metabolites; (**C**) Regulatory effects of plasma metabolites on IA.

Mendelian randomization analysis revealed a significant positive causal relationship between F2R and IA in Figure 5A. Figure 5B illustrated F2R’s regulatory effects on plasma metabolites. It positively regulated three metabolites: xanthurenate levels (OR = 1.2164, 95% CI: 1.0181–1.4533, *p* = 0.0310), dopamine 3-O-sulfate levels (OR = 1.3893, 95% CI: 1.1173–1.7275, *p* = 0.0031), and the sphingosine-to-phosphate ratio (OR = 1.2357, 95% CI: 1.0326–1.4788, *p* = 0.0209). Conversely, it negatively regulated five metabolites: phenylacetylglutamine levels (OR = 0.7805, 95% CI: 0.6604–0.9225, *p* = 0.0037), cinnamoylglycine levels (OR = 0.8030, 95% CI: 0.6730–0.9580, *p* = 0.0149), heptenedioate (C7:1-DC) levels (OR = 0.7753, 95% CI: 0.6522–0.9215, *p* = 0.0039), the mannose-to-S-methylcysteine ratio (OR = 0.7847, 95% CI: 0.6570–0.9372, *p* = 0.0075), and the hypotaurine-to-cysteine ratio (OR = 0.7999, 95% CI: 0.6723–0.9519, *p* = 0.0119). Figure 5C confirmed the causal effects of all eight metabolites on IA. The results were as follows: Xanthurenate levels (OR = 0.5986, 95% CI: 0.4364–0.9975, *p* = 0.0486), Phenylacetylglutamine levels (OR = 0.5212, 95% CI: 0.3031–0.8961, *p* = 0.0184), Cinnamoylglycine levels (OR = 0.6503, 95% CI: 0.4471–0.9459, *p* = 0.0244), and Hypotaurine to cysteine ratio (OR = 0.6543, 95% CI: 0.4397–0.9735, *p* = 0.0364) exhibited significant negative effects on IA risk. Conversely, the following metabolites showed significant positive effects: Dopamine 3-O-sulfate levels (OR = 1.7538, 95% CI: 1.1126–2.7646, *p* = 0.0155), Heptenedioate (C7:1-DC) levels (OR = 1.8552, 95% CI: 1.0201–3.3739, *p* = 0.0429), Sphingosine to phosphate ratio (OR = 1.6578, 95% CI: 1.0077–2.7273, *p* = 0.0466), and Mannose to S-methylcysteine ratio (OR = 1.7507, 95% CI: 1.1069–2.7690, *p* = 0.0166).

These findings outline a key regulatory pathway involving plasma metabolites, in which F2R is crucial for promoting IA development. We employed a two-step MR approach. First, we assessed the causal effect of F2R genetic instruments on mediator variables, identifying eight significant MR results from 1400 mediator outcomes (Supplementary Table S6). In the second step, we evaluated the causal impact of these eight significant plasma metabolites on IA risk (Supplementary Table S7). Three metabolites showed significant mediation proportions: phenylacetylglutamine levels (β_1_ × β_2_/β__all_ = 0.157), dopamine 3-O-sulfate levels (β_1_ × β_2_/β__all_ = 0.180), and the sphingosine-to-phosphate ratio (β_1_ × β_2_/β__all_ = 0.104) (Supplementary Table S8).

This study validates F2R’s crucial role in IA pathogenesis and highlights its regulatory influence on plasma metabolites. The two-step MR analysis provides mechanistic insights into how F2R mediates IA risk through metabolic pathways, offering novel perspectives and potential therapeutic targets for IA prevention and treatment.

### 3.8. miRNA-Mediated Effects on the Risk of IA Through the F2R

**Figure 6:**
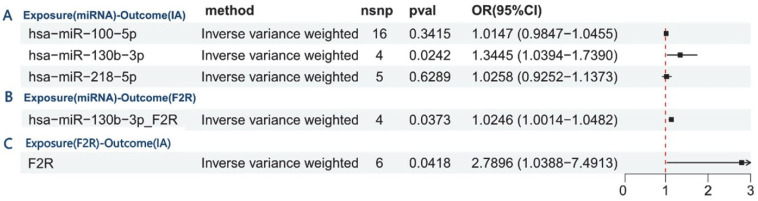
Mediation analysis and Mendelian randomization reveal F2R’s mediating role between miR-130b-3p and IA. (**A**) Causal effect of miR-130b-3p on IA risk. (**B**) miR-130b-3p upregulates F2R expression (positive effect). (**C**) Confirmed causal relationship between F2R and IA, validating prior findings.

This study employed mediation analysis and MR to investigate the role of F2R as a mediator between miR-130b-3p and IA. Figure 6A shows a significant positive relationship between miR-130b-3p and IA. Figure 6B further shows the positive effect of miR-130b-3p on F2R. Figure 6C confirms the causal relationship between F2R and IA, corroborating prior findings. these analyses found no evidence of horizontal pleiotropy or heterogeneity.

These findings reveal a key regulatory axis mediated by F2R, in which miR-130b-3p plays a pivotal role in promoting IA development. This pathway illustrates a three-step mechanism: first, miR-130b-3p regulates F2R (β_1_ = 0.024), which increases coagulation receptor expression; second, F2R increases the risk of IA (β_1_ = 1.026), raising disease susceptibility; finally, the positive relationship between miR-130b-3p and IA (β__all_ = 0.296) shows that F2R accounts for 8.4% of the effect (β_1_ × β_2_/β__all_ = 0.084), highlighting the biological importance of F2R in the pathogenic mechanism of miR-130b-3p (Supplementary Tables S9 and S10).

## 4. Discussion

This study explores the potential role of PBDE-47 exposure in the development of IA through metabolic and epigenetic mechanisms. Human exposure to PBDE-47 occurs mainly via ingestion of contaminated house dust (a significant route in children), dietary intake of lipid-rich animal products (due to its lipophilicity and bioaccumulation potential), inhalation of contaminated air, and transplacental or breastfeeding-mediated early-life exposure, making it a widespread environmental concern. We identified 15 molecular targets associated with both PBDE-47 exposure and IA pathogenesis, among which F2R emerged as a potential mediator. These findings imply that PBDE-47 may disrupt vascular function and influence IA risk through metabolic pathways, underscoring the need for further investigation into its long-term vascular effects.

Our results are consistent with prior studies linking PBDEs to cardiovascular impairment, especially via detrimental effects on vascular endothelial cells. For example, Berghuis et al. (2022) reported that elevated prenatal PBDE levels were correlated with increased triglyceride levels in adolescents, suggesting a metabolic pathway for cardiovascular risk [43]. Similarly, Hou et al. (2019) demonstrated that BDE-209 exposure triggered autophagy and apoptosis in human umbilical vein endothelial cells (HUVECs) through the IRE1α/Akt/mTOR pathway, indicating direct endothelial toxicity [44]. These studies reinforce our conclusion that PBDEs compromise endothelial integrity and function.

However, some reports present conflicting mechanistic insights. A systematic review by Chen et al. (2023) acknowledged the association between PBDEs and cardiovascular risk but emphasized uncertainty regarding underlying pathways and individual variability in response [45]. Wang et al. (2025) found no significant link between PBDEs and blood lipid levels in adults, implying possible modulation by genetic or environmental factors [46]. These discrepancies may arise from differences in study design, population characteristics, and specific PBDE congeners examined, highlighting the need for further mechanistic studies.

Overall, our study supports the notion that PBDE exposure harms cardiovascular health, particularly via endothelial dysfunction. Yet, inconsistent findings across studies necessitate deeper investigation into the precise mechanisms governing PBDE-related cardiovascular effects.

A key finding is that F2R serves as a pivotal mediator of IA risk by regulating plasma metabolites, illustrating how chemical exposure may lead to disease [47]. Our MR analysis supports a significant causal effect of F2R on IA, mediated through positive regulation of xanthurenate and dopamine 3-O-sulfate, and negative regulation of phenylacetylglutamine and the hypotaurine-to-cysteine ratio—each implicated in IA pathogenesis [48].

This regulatory network suggests that F2R mediates chemical-induced IA risk by reshaping metabolic profiles. Specifically, PBDE-47 may alter F2R expression through epigenetic mechanisms such as miRNA dysregulation. Evidence suggests that environmental pollutants can alter miRNA expression [49,50] and these miRNAs have been shown to positively regulate F2R. Prior research has indicated that miR-130b-3p is instrumental in various biological functions, including cell growth, programmed cell death, and metabolic control [51,52,53]. Its biological functions are mainly realized by targeting and regulating specific genes [51,52,54]. miR-130b-3p plays an important role in various biological processes, and research on its regulatory mechanisms and targets provides important clues for understanding its function in diseases [55,56]. This forms a feedback loop that exacerbates IA risk. Exposure to PBDE-47 may elevate miR-130b-3p levels, which in turn upregulates F2R, leading to disruption of endothelial function and metabolic homeostasis.

Abnormal plasma metabolite levels have been observed in IA patients, distinguishing ruptured from unruptured cases and underscoring the role of metabolic dysregulation in disease progression [57,58]. Environmental chemicals can perturb metabolic processes [59], promoting the accumulation of harmful metabolites conducive to IA.

Our study also highlights the interplay between chemical exposure and genetic susceptibility. Genetic background can modulate individual responses to toxins, as demonstrated in populations with varying genetic risks exposed to environmental chemicals [58]. Thus, an integrated approach considering both genetic and environmental factors is essential for understanding IA pathogenesis.

In conclusion, our findings position F2R as a central mediator in IA development via metabolic reprogramming, driven by chemical exposure and modulated by epigenetic mechanisms. These results offer new insights for therapeutic strategies aimed at mitigating IA risk through metabolic intervention and chemical exposure control.

This study employs an MR framework, strengthening causal inference over conventional observational studies. By integrating genetic data and applying robust MR methods (including IVW and MR-Egger regression), we reduce confounding and enhance validity. Sensitivity analyses—such as leave-one-out testing and pleiotropy evaluation—support the consistency and reliability of our results. This approach provides a model for future studies in genetic epidemiology.

## 5. Conclusions

This research identifies a significant causal link between different plasma proteins, especially F2R, and the likelihood of developing IA. It underscores the possible involvement of metabolic pathways and the regulatory influence of miRNAs in this condition. However, our analysis is subject to several limitations. Firstly, the absence of wet lab validation to support the observed associations. Secondly, a relatively small sample size that may restrict the generalizability of our findings, and a lack of clinical validation to confirm these results in a patient cohort. Additionally, the integration of multiple datasets may introduce batch effects, potentially impacting the consistency of the results. Moreover, we were unable to estimate the population-level risk of IA attributable to PBDE-47 exposure. The IA GWAS data used in this study includes both unruptured and ruptured cases, which makes it difficult to isolate associations specifically related to rupture events. Furthermore, although MR estimates the effects of lifelong genetic exposure, it does not provide information about the dose–response relationship or key time windows of PBDE-47 exposure. Additionally, since the genetic data used in our MR analysis are primarily from European populations, this could introduce population structure bias, limiting the generalizability of our findings to other ethnic groups. Future prospective cohort studies with repeated exposure measurements will help address these important issues.

In summary, our research highlights the essential function of F2R in the pathogenesis of IA and its impact on plasma metabolites, providing novel insights and potential therapeutic targets for the prevention and management of IA. Further investigations are necessary to corroborate these findings through clinical trials and to examine the underlying biological mechanisms more thoroughly, thereby achieving a holistic comprehension of the interconnections among plasma proteins, metabolites, and the risk of IA.

## Figures and Tables

**Table 1:** Binding Energies of PBDE-47 with Various Target Proteins.

Ligand	PDB Identifier	Target	Affinity, kcal/mol
PBDE-47	8hnk	CXCL11	−5.769
	3vw7	F2R	−5.516
	5l0s	F7	−7.762
	1ev2	FGFR2	−5.961
	6u1u	ANGPTL4	−6.051
	AF-Q76M96	CCDC80	−4.841
	7ev1	CDH17	−5.803
	4oel	CTSC	−5.747
	9gj2	CTSS	−7.066
	AF-P12034	FGF5	−4.315
	6u66	ADIPOQ	−6.469
	1dgf	CAT	−5.717
	2uur	COL9A1	−5.081
	3ehu	CRH	−7.036
	5gw9	CSF3	−6.227

**Table 2:** Binding free energies and energy components predicted by MM/GBSA (kcal/mol).

System Name	F2R_PBDE-47
ΔE_vdw_	−49.21 ± 2.42
ΔE_elec_	−11.62 ± 2.09
ΔG_GB_	27.98 ± 2.37
ΔG_SA_	−4.98 ± 0.08
ΔG_bind_	−37.85 ± 3.12

ΔE_vdW_: van der Waals energy. ΔE_elec_: electrostatic energy. ΔG_GB_: electrostatic contribution to solvation. ΔG_SA_: non-polar contribution to solvation. ΔG_bind_: binding free energy.

## Data Availability

The original data used in this study are publicly available from the following sources: Plasma protein genome-wide association study (GWAS) data from the UK Biobank-Proteomics Project (UKB-PPP) are accessible under DOIs: 10.1038/s41586-023-06547-x, 10.1038/s41586-023-06563-x, and 10.1038/s41586-023-06592-6. Plasma metabolite GWAS data for 1400 metabolites are available from the GWAS Catalog (accession range: GCST90199621–GCST90201020) under DOI: 10.1038/s41588-022-01270-1. In-tracranial aneurysm (IA) GWAS data are accessible under accession GCST90044003 and DOI: 10.1038/s41588-021-00954-4. miRNA expression quantitative trait loci (eQTL) data are available under DOI: 10.1038/ncomms7601.

## Supplementary Materials

The following supporting information can be downloaded at: https://www.mdpi.com/article/10.3390/brainsci15101091/s1. Table S1. Data sources of exposures, mediators and outcome. Table S2. 1400 metabolites included in the study. Table S3. Associations between UKB-PPP and intracranial aneurysms. Table S4. Associations between intracranial aneurysms and UKB-PPP. Table S5. Results of SMR and HEIDI test between circulating protains and intracranial aneurysms. Table S6. Associations between UKB-PPP and Plasma metabolites. Table S7. Associations between Plasma metabolites and intracranial aneurysms. Table S8. Plasma metabolites Mediation effect. Table S9. Summary of the Regulatory Axis Mediated by F2R in the Pathogenesis of IA. Table S10. F2R Mediation effect. Figure S1. Forest Plot of Causal Associations Between Plasma Proteins and Intracranial Aneurysm Risk.
