# Supplementary figures and images for: Multi-Omics Integration Reveals PBDE-47 as an Environmental Risk Factor for Intracranial Aneurysm via F2R-Mediated Metabolic and Epigenetic Pathways
Source: Brain Sci. 2025 Oct 9;15(10):1091. doi: 10.3390/brainsci15101091 (PMC12564836; doi:10.3390/brainsci15101091)

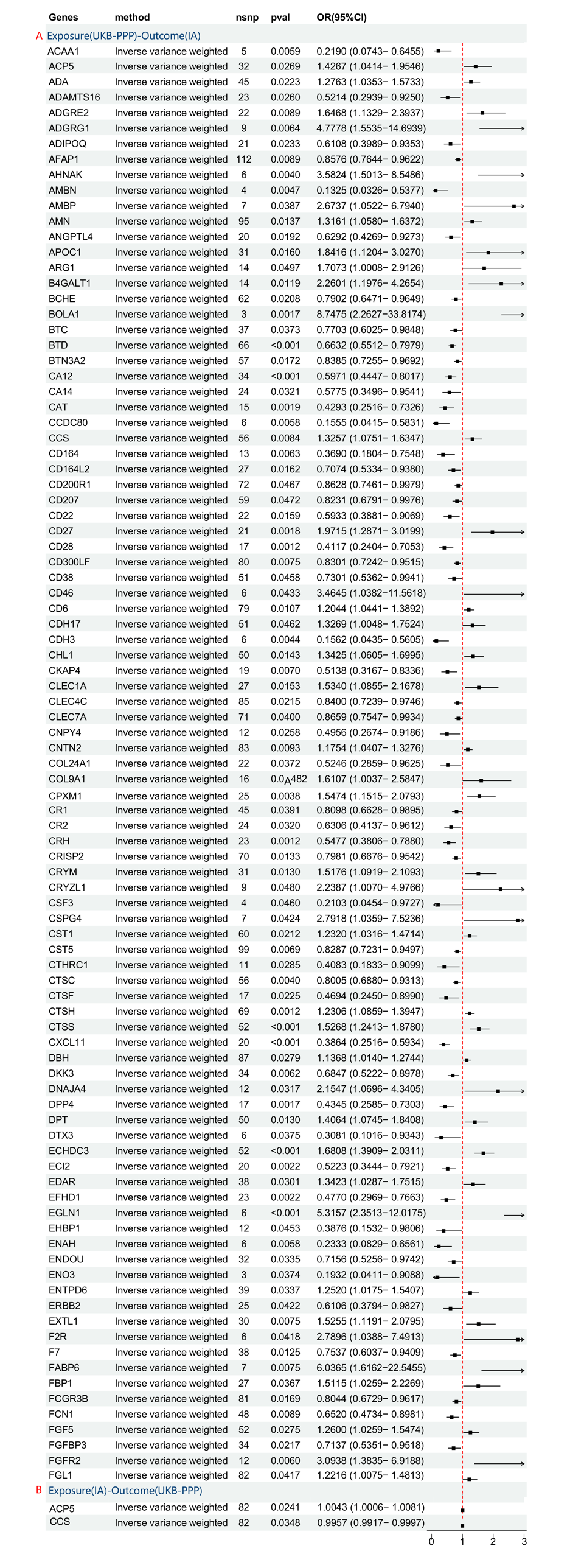

Supplement: Supplementary file 1 [file brainsci-15-01091-s001.zip › brainsci-3794673-Supplementary Figure S1.tif]
